# Equilibrium Wigner Function for Fermions and Bosons in the Case of a General Energy Dispersion Relation

**DOI:** 10.3390/e22091023

**Published:** 2020-09-13

**Authors:** Vito Dario Camiola, Liliana Luca, Vittorio Romano

**Affiliations:** Department of Mathematics and Computer Science, University of Catania, 95125 Catania, Italy; lilianaluca88@gmail.com (L.L.); romano@dmi.unict.it (V.R.)

**Keywords:** Wigner function, quantum entropy, transport of bosons and fermions

## Abstract

The approach based on the Wigner function is considered as a viable model of quantum transport which allows, in analogy with the semiclassical Boltzmann equation, to restore a description in the phase-space. A crucial point is the determination of the Wigner function at the equilibrium which stems from the equilibrium density function. The latter is obtained by a constrained maximization of the entropy whose formulation in a quantum context is a controversial issue. The standard expression due to Von Neumann, although it looks a natural generalization of the classical Boltzmann one, presents two important drawbacks: it is conserved under unitary evolution time operators, and therefore cannot take into account irreversibility; it does not include neither the Bose nor the Fermi statistics. Recently a diagonal form of the quantum entropy, which incorporates also the correct statistics, has been proposed in Snoke et al. (2012) and Polkovnikov (2011). Here, by adopting such a form of entropy, with an approach based on the Bloch equation, the general condition that must be satisfied by the equilibrium Wigner function is obtained for general energy dispersion relations, both for fermions and bosons. Exact solutions are found in particular cases. They represent a modulation of the solution in the non degenerate situation.

## 1. Introduction

The use of the Wigner function is one of the way to study quantum transport. Its main advantage is that a description similar to the classical or semiclassical transport is obtained in a suitable phace-space. For example the mean values are expectation values with respect to the Wigner function as it would be a probability density. Moreover, the semiclassical limit of the Wigner transport equation recovers, at least formally, the Boltzmann transport one. There is a huge body of literature regarding the Wigner equation and the way to numerically solve it (see for example [1,2,3] and references therein). However, the most part of the works in the subject consider a quadratic dispersion relation for the energy. Instead, for several material like semiconductors or semimetal, e.g., graphene, other dispersion relations must be considered [4,5,6,7]. From the Wigner transport equation quantum hydrodynamical models have been obtained in [8] for charge transport in silicon in the case of parabolic bands, while in [9] the same has been devised for electrons moving in graphene.

A starting point is the determination of the equilibrium Wigner function. It can be obtained by using the Jaynes approach [6,10,11,12] of maximizing the entropy under suitable constraints on the expectation values. A crucial issue is the expression of the entropy in the quantum case. In [8,9] the standard prescription proposed by von Neumann has been adopted but it leads to a semiclassical limit represented by the Maxwell–Boltzmann distribution. For dilute gases this can constitute an acceptable approximation but for strongly degenerate gases it is necessary to include the appropriate statistics as for example done in [13] where expansions of the equilibrium Wigner functions were determined by using the Moyal formalism.

Moreover, in a closed system the von Neumann entropy is conserved (see the papers [14,15]) because the evolution is described by unitary operators. Arguing on such a remark, in [15] it has suggested to use as entropy only the diagonal contribution and has proved that it increase in time according to the second law of thermodynamics.

Here, similarly to what already considered in [10] and more recently pointed out in [14] and employed in [13], we adopt a diagonal form of the entropy which incorporates also the correct statistics but we use an approach based on the Bloch equation to get the equilibrium Wigner function for a general dispersion relation. Both the case of fermions and bosons are treated. The general solution is very difficult to get analytically and a numerical approach is the only viable approach. Here, exact solutions are obtained in particular cases. They represent a modulation of the solution in the non degenerate situation.

The plan of the paper is as follows. In Section 2 the general features of the quantum transport based on the Wigner function are recalled. In Section 3 the general problem of determining the equilibrium density matrix is tackled while in Section 4 the general equation for determining the equilibrium Wigner equation is deduced. In the last section examples of solutions in the spatially homogeneous case are given both for fermions and bosons. Some details are postponed in the Appendix A.

## 2. Quantum Transport Based on the Wigner Equation

Let us introduce the single-particle density matrix, ρ(r,s,t) which is related to the wave function ψ by

$$\rho \left(r,s,t\right)=\psi \left(r,t\right)\bar{\psi }\left(s,t\right)\;\mathrm{for}\;\mathrm{any}\;r,s\in R^{d},$$

with *d* dimension of the space, e.g., for electrons flowing in graphene d=2 while for charge carriers in bulk silicon d=3. It satisfies the relation

ρ(r,r,t)=n(r,t),

where n(r,t) is the position probability density.

The time evolution of the density matrix is described by the quantum Liouville equation

$$i\hbar \frac{\partial }{\partial t}\rho \left(r,s,t\right)=\left(H_{r}-H_{s}\right)\rho \left(r,s,t\right)$$

where $H_{r}$ and $H_{s}$ represent the symbols of Hamiltonians acting with respect to the r and s variable respectively.

If E(p) is the energy band in terms of the crystal momentum p=ℏk, the symbol of the Hamiltonian reads

H(r,p)=E(p)+Φ(r,t)

where the external potential Φ(r,t) here is assumed to be real. Moreover, we assume that E(p) is a even function of the modulus of p.

On account of the quantum mechanics correspondence principle $p⟶-i\hbar \nabla_{r}$, the quantum Liouville equation reads

(1) $$i\hbar \frac{\partial }{\partial t}\rho \left(r,s,t\right)=\left(E\left(-i\hbar \nabla_{r}\right)-E\left(-i\hbar \nabla_{s}\right)\right)\rho \left(r,s,t\right)+\left[(\Phi (r,t)-\Phi (s,t))\right]\rho \left(r,s,t\right).$$

Given a function $g\in L^{1}\left(R^{d}\right)$ let us denote by F[g](η) its Fourier transform

$$F\left[g\right]\left(\eta \right)=\int_{R_{v}^{d}}g\left(v\right)e^{-iv\cdot \eta }dv,$$

and let us denote by $F^{-1}$ the inverse Fourier transform

$$F^{-1}\left[h\left(\eta \right)\right]=\frac{1}{{\left(2\pi \right)}^{d}}\int_{R_{\eta }^{d}}h\left(\eta \right)e^{iv\cdot \eta }d\eta .$$

with h(η)=F[g](η).

In order to derive a transport equation, let us introduce the single particle Wigner quasi-distribution w(x,p,t), depending on the position x, momentum p and time *t*, defined as

$$w\left(x,p,t\right)=F\left[\rho \left(x+\frac{y}{2},x-\frac{y}{2},t\right)\right]\left(x,p,t\right)=\int_{R_{y}^{2}}\rho \left(x+\frac{y}{2},x-\frac{y}{2},t\right)e^{-ip\cdot y/\hbar }dy.$$

That satisfies the following equation obtained from (1) by a Wigner transformation (see Appendix A for the details)

(2) $$\frac{\partial w(x,p,t)}{\partial t}+S\left[E\right]w\left(x,p,t\right)+\theta \left[E\right]w\left(x,p,t\right)=C\left[w\right].$$

which is the quantum counterpart of the semiclassical Boltzmann transport equation and sometimes is referred to the Wigner–Boltzmann equation.

S[E] and θ[E] represent the pseudo-differential operators

(3) $$S\left[E\right]w\left(x,p,t\right)=\frac{i}{\hbar {\left(2\pi \right)}^{d}}\int_{R_{x^{\prime }}^{d}\times R_{\nu }^{d}}\left[E\left(p+\frac{\hbar }{2}\nu ,t\right)-E\left(p-\frac{\hbar }{2}\nu ,t\right)\right]w\left(x^{\prime },p,t\right)e^{-i(x^{\prime }-x)\cdot \nu }dx^{\prime }d\nu ,$$

(4) $$\theta \left[E\right]w\left(x,p,t\right)=\frac{i}{\hbar {\left(2\pi \right)}^{d}}\int_{R_{p^{\prime }}^{d}\times R_{\eta }^{d}}\left[\Phi \left(x+\frac{\hbar }{2}\eta ,t\right)-\Phi \left(x-\frac{\hbar }{2}\eta ,t\right)\right]w\left(x,p^{\prime },t\right)e^{i(p^{\prime }-p)\cdot \eta }dp^{\prime }d\eta .$$

and C[w] is a sort of collision term.

In realistic cases the expression of C[w] is rather complex (see for example [1]). A simplified model is based on the relaxation time approximation

$$C\left[w\right]=-\nu \left(w-w_{eq}\right).$$

where $w_{eq}$ is the equilibrium Wigner function and ν plays the role of a collision frequency which in general can depend on p. Moreover, several hydrodynamical models introduce quantum corrections just from the equilibrium Wigner function, see for example [8,16]. Therefore, a crucial step is to find out the expression of $w_{eq}$.

## 3. Equilibrium Density Function

Let us denote with $\hat{\rho }$ the density matrix operator. It is related to ρ by

$$\left(\hat{\rho }\;\phi \right)\left(x,t\right)=\int_{R^{2}}\rho \left(x,y,t\right)\phi \left(y\right)dy,$$

for any suitable test function ϕ. In other words, ρ(x,y,t) is the kernel of $\hat{\rho }$. The latter solves the operatorial quantum Liouville equation

$$i\hbar \frac{\partial }{\partial \;t}\hat{\rho }=\left[\hat{H},\hat{\rho }\right],$$

where $\hat{H}$ is the Hamiltonian and $\left[\hat{H},\hat{\rho }\right]=\hat{H}\hat{\rho }-\hat{\rho }\hat{H}$ is the commutator. In a steady state, and in particular at equilibrium, $\frac{\partial }{\partial t}\hat{\rho }=0$ and therefore $[\hat{H},\hat{\rho }]=0$.

The equilibrium density matrix can be obtained by employing a generalisation of the Maximum Entropy Principle (hereafter MEP) in a quantum context [10,17,18] (for the semiclassical case see [6,9,12,19,20,21,22]). According to the quantum version of MEP the equilibrium density matrix is obtained by maximising the quantum entropy $S_{q}$ under suitable constraints on the expectation values.

If we consider particles moving in a thermal bath, e.g., electrons in a semiconductor keeping phonons at equilibrium with temperature *T*, the equilibrium density matrix has to satisfy the constraints

(5) $$\begin{array}{c} \mathrm{tr}\hat{\rho }=1,\;<\hat{H}>=\mathrm{tr}\left(\hat{\rho }\hat{H}\right), \end{array}$$

where tr is the trace operator. Since at equilibrium $\hat{H}$ commutes with $\hat{\rho }$, there exists an orthonormal basis such that both $\hat{H}$ and $\hat{\rho }$ have a diagonal representation (for the sake of simplicity we assume that the spectrum is discrete)

$$\hat{H}=\sum_{i}E_{i}|\psi_{i}><\psi_{i}|,\;\hat{\rho }=\sum_{i}\rho_{i}\left|\psi_{i}><\psi_{i}\right|.$$

A controversial question is the appropriate expression of $S_{q}$. The standard formulation is that of von Neumann [23]

(6) $$\begin{array}{c} S_{q}=-k_{B}\;\mathrm{tr}\left(\hat{\rho }\ln\hat{\rho }\right), \end{array}$$

which is the natural generalization to a quantum context of the Maxwell–Boltzmann one or, in information theory, the generalization of the Shannon entropy. Maximizing the entropy (6) under the constraints (5) means maximizing the objective function

$$-\sum_{i}k_{B}\;\rho_{i}\ln\rho_{i}+\alpha \left(1-\sum_{i}\rho_{i}\right)+\beta \left(<\hat{H}>-\sum_{i}\rho_{i}E_{i}\right),$$

with α and β Lagrange multipliers. One gets, after an obvious renormalization of the Lagrange multipliers,

(7) $$\begin{array}{c} \rho_{i}=\exp\left(-\beta (E_{i}-\phi_{F}))\right) \end{array}$$

where $\phi_{F}$ is the quasi-Fermi potential and $\beta =\frac{1}{k_{B}T}$.

Therefore, the equilibrium density matrix operator is given by the following. We recall that if $\hat{A}$ is a Hermitian operator and *f* a function regular enough, $f(\hat{A})$ is defined as follows. In a basis where $\hat{A}$ has a diagonal representation

$$\hat{A}=\sum_{i}a_{i}\left|\psi_{i}><\psi_{i}\right|,$$

with $a_{i}$ eigenvalues of $\hat{A}$, we set

$$f\left(\hat{A}\right)=\sum_{i}f\left(a_{i}\right)\left|\psi_{i}><\psi_{i}\right|,$$

provided $f(a_{i})$ makes sense.

$$\begin{array}{c} {\hat{\rho }}_{eq}=\exp\left(-\beta \left(\hat{H}-\phi_{F}\right)\right), \end{array}$$

which appears as the natural counterpart of the Maxwell–Boltzmann density.

Now, we have to face two important problem related to the von Neumann entropy. In case of fermions or bosons the equilibrium Wigner function does not include the Fermi or the Bose statistics. Moreover (see the papers [14,15]), in a closed system $S_{q}$ is conserved because the evolution of the system is described by unitary operators. However, in a open system, like a semiconductor electron device, there is a fast decay of the off-diagonal terms and practically only the diagonal contribution to $S_{q}$ survives. Arguing on such a remark, Polkovnikov [15] has suggested to use as entropy only the diagonal contribution $S_{d}$ and has proved that it increase in time according to the second law of thermodynamics. A further modification, already considered in [10], more recently pointed out in [14] and employed in [13], which incorporate also the correct statistics is to define the quantum entropy as

(8) $$\begin{array}{c} S=-k_{B}\sum_{k}\left[\rho_{kk}\ln\rho_{kk}\mp \left(1\pm \rho_{kk}\right)\ln\left(1\pm \rho_{kk}\right)\right], \end{array}$$

where the $\rho_{kk}$’s are the expectation values of the diagonal elements of $\hat{\rho }$ which can be interpreted as occupation numbers, the upper sign being valid for bosons and the lower one for fermions. This could solve the problem of the limit of the equilibrium Wigner function even if the needed calculations become much more involved to carry out analytically [13].

Maximizing the entropy (8) under the constraints (5) means maximizing the objective function

$$-k_{B}\;\sum_{k}\left[\rho_{kk}\ln\rho_{kk}\mp \left(1\pm \rho_{kk}\right)\ln\left(1\pm \rho_{kk}\right)\right]+\alpha \left(1-\sum_{k}\rho_{kk}\right)+\beta \left(<H>-\sum_{k}\rho_{kk}E_{kk}\right),$$

with α and β Lagrange multipliers. One gets, after an obvious renormalization of the Lagrange multipliers,

$$\begin{array}{c} \rho_{kk}=\frac{1}{\exp\left(\beta (E_{kk}-\phi_{F}))\pm 1\right)} \end{array}$$

where again $\phi_{F}$ is the quasi-Fermi potential and $\beta =\frac{1}{k_{B}T}$.

Therefore, the equilibrium density matrix operator is given by

(9) $$\begin{array}{c} {\hat{\rho }}_{eq}=\frac{1}{\exp\left(\beta (\hat{H}-\phi_{F})\right)\pm 1} \end{array}$$

## 4. Equilibrium Wigner Function

Once the equilibrium density function has been determined, the equilibrium Wigner function can be obtained by a direct evaluation of the following Fourier transform

$$w_{eq}\left(x,p,t\right)=F\left[\rho_{eq}\left(x+\frac{y}{2},x-\frac{y}{2},t\right)\right]\left(x,p,t\right)=\int_{R_{y}^{d}}\rho_{eq}\left(x+\frac{y}{2},x-\frac{y}{2},t\right)e^{-ip\cdot y/\hbar }dy.$$

However, explicit expressions are difficult to find out and in the most part they have been obtained only in the case of the free particle energy dispersion relation. Usually an expansion based on the Moyal calculus is employed (see for example [13]). Here, we adopt an alternative approach. We will write down a differential equation which must be satisfied by the equilibrium Wigner function.

By taking the derivative of (9) with respect to β, one has

$$\frac{\partial {\hat{\rho }}_{eq}}{\partial \beta }=-\frac{1}{2}\left[\left(\hat{H}-\phi_{F}\right){\hat{\rho }}_{eq}+\left(\hat{H}-\phi_{F}\right){\hat{\rho }}_{eq}\right]\pm \frac{1}{2}\left[\left(\hat{H}-\phi_{F}\right){\hat{\rho }}_{eq}^{2}+\left(\hat{H}-\phi_{F}\right){\hat{\rho }}_{eq}^{2}\right]$$

where the commutation relation between $\hat{H}$ and ${\hat{\rho }}_{eq}$ has been used. For any suitable test function φ, we have

$$\begin{array}{cc} & \int_{R^{2}}\frac{\partial \;\rho_{eq}\left(r,s,\beta \right)}{\partial \;\beta }\varphi \left(s\right)ds=-\frac{1}{2}\int_{R^{2}}[H_{r}\rho_{eq}\left(r,s,\beta \right)+H_{s}\rho_{eq}\left(r,s,\beta \right)+\\ \mp & H_{r}\rho_{eq}^{2}\left(r,s,\beta \right)\mp H_{s}\rho_{eq}^{2}\left(r,s,\beta \right)-2\phi_{F}\rho_{eq}\left(r,s,\beta \right)\pm 2\phi_{F}\rho_{eq}^{2}\left(r,s,\beta \right)]\varphi \left(s\right)ds. \end{array}$$

From general considerations in quantum mechanics, we require that $\hat{H}$ must be self-adjoint

$$\int_{R^{2}}\rho_{eq}\left(r,s,\beta \right)H_{s}\phi \left(s\right)\;ds=\int_{R^{2}}H_{s}\rho_{eq}\left(r,s,\beta \right)\phi \left(s\right)\;ds$$

and therefore from the previous relations we get

$$\begin{array}{ccc} \frac{\partial \;\rho_{eq}\left(r,s,\beta \right)}{\partial \beta } & = & -\frac{1}{2}\left[H_{r}\rho_{eq}\left(r,s,\beta \right)+H_{s}\rho_{eq}\left(r,s,\beta \right)\right]\\ & \pm & \frac{1}{2}\left[H_{r}\rho_{eq}^{2}\left(r,s,\beta \right)+H_{s}\rho_{eq}^{2}\left(r,s,\beta \right)\right]+\phi_{F}\rho_{eq}\left(r,s,\beta \right)\mp \phi_{F}\rho_{eq}^{2}\left(r,s,\beta \right), \end{array}$$

which is named Bloch equation

Fourier transforming the Bloch equation, one finds the following equation for the equilibrium Wigner function

(10) $$\begin{array}{c} \frac{\partial w_{eq}\left(x,k,\beta \right)}{\partial \beta }=-\frac{1}{2}F[\left(\varepsilon \left(p+\frac{\hbar }{2}\nu \right)+\varepsilon \left(p-\frac{\hbar }{2}\nu \right)\right)u\left(x,y,\beta \right)\\ +\left(\Phi \left(x+\frac{1}{2}y\right)+\Phi \left(x-\frac{1}{2}y\right)\right)u\left(x,y,\beta \right)]\left(x,k,\beta \right)\pm \frac{1}{2}F[\left(\varepsilon \left(p+\frac{\hbar }{2}\nu \right)+\varepsilon \left(p-\frac{\hbar }{2}\nu \right)\right)u^{2}\left(x,y,\beta \right)\\ +\left(\Phi \left(x+\frac{1}{2}y\right)+\Phi \left(x-\frac{1}{2}y\right)\right)u^{2}\left(x,y,\beta \right)]\left(x,k,\beta \right)+\Phi_{F}\;w_{eq}\left(x,k,\beta \right)\\ \mp \Phi_{F}\;F\left[u^{2}\left(x,y,\beta \right)\right]\left(x,k,\beta \right). \end{array}$$

Since for β=0 we must have ${\hat{\rho }}_{eq}=1$, it follows

$$\int_{R^{d}}\rho \left(r,s,0\right)\phi \left(s\right)ds=\phi \left(r\right),$$

which implies (δ(r) denotes the Dirac distribution)

ρ(r,s,0)=δ(s−r),

wherefrom the condition

(11) $$w_{eq}\left(x,p,0\right)=1.$$

Equation (10) augmented with the initial condition (11) is the basic relation of this work. It represents the general condition that the equilibrium Wigner function has to satisfy for a general dispersion relations in the degenerate case.

Equation (10) is very difficult to tackle analytically. A form more amenable to sought for analytical solutions is obtained by introducing the following approximations up to first order in $\hbar^{2}$

$$\begin{array}{ccc} \varepsilon \left(p+\frac{\hbar }{2}\nu \right)+\varepsilon \left(p-\frac{\hbar }{2}\nu \right) & \approx & 2\varepsilon \left(p\right)+\frac{\hbar^{2}}{4}\frac{\partial^{2}\varepsilon }{\partial p_{i}\partial p_{j}}\nu_{i}\nu_{j}=2\varepsilon \left(p\right)+\frac{1}{4}\frac{\partial^{2}\varepsilon }{\partial k_{i}\partial k_{j}}\nu_{i}\nu_{j},\\ \Phi \left(x+\frac{\hbar }{2}\eta \right)+\Phi \left(x-\frac{\hbar }{2}\eta \right) & \approx & 2\Phi \left(x\right)+\frac{\hbar^{2}}{4}\frac{\partial^{2}\Phi }{\partial x_{i}\partial x_{j}}\eta_{i}\eta_{j}=2\Phi \left(x\right)+\frac{1}{4}\frac{\partial^{2}\Phi }{\partial x_{i}\partial x_{j}}y_{i}y_{j}. \end{array}$$

After substituting into (10), one obtains

(12) $$\begin{array}{c} \frac{\partial w_{eq}\left(x,k,\beta \right)}{\partial \beta }=-\varepsilon \left(p\right)\;w_{eq}\left(x,k,\beta \right)+\frac{1}{8}\frac{\partial^{2}\varepsilon }{\partial k_{i}\partial k_{j}}\frac{\partial^{2}w_{eq}}{\partial x_{i}\partial x_{j}}+\\ -\;\Phi \left(x\right)\;w_{eq}\left(x,k,\beta \right)+\frac{1}{8}\frac{\partial^{2}\Phi }{\partial x_{i}\partial x_{j}}\frac{\partial^{2}w_{eq}\left(x,k,\beta \right)}{\partial k_{i}\partial k_{j}}\\ \pm \frac{1}{{\left(2\pi \right)}^{d}}\;\varepsilon \left(p\right)\int_{R^{2}}w_{eq}\left(x,k^{\prime },\beta \right)w_{eq}\left(x,k^{\prime }-k,\beta \right)dk^{\prime }\\ \mp \frac{1}{{\left(2\pi \right)}^{d}}\frac{1}{8}\frac{\partial^{2}\varepsilon }{\partial k_{i}\partial k_{j}}\frac{\partial^{2}}{\partial x_{i}\partial x_{j}}\int_{R^{2}}w_{eq}\left(x,k^{\prime },\beta \right)w_{eq}\left(x,k^{\prime }-k,\beta \right)dk^{\prime }\\ \pm \frac{1}{{\left(2\pi \right)}^{d}}\;\Phi \left(x\right)\int_{R^{2}}w_{eq}\left(x,k^{\prime },\beta \right)w_{eq}\left(x,k^{\prime }-k,\beta \right)dk^{\prime }\\ \mp \frac{1}{{\left(2\pi \right)}^{d}}\frac{1}{8}\frac{\partial^{2}\Phi }{\partial x_{i}\partial x_{j}}\frac{\partial^{2}}{\partial k_{i}\partial k_{j}}\int_{R^{2}}w_{eq}\left(x,k^{\prime },\beta \right)w_{eq}\left(x,k^{\prime }-k,\beta \right)dk^{\prime }+\Phi_{F}\;w_{eq}\left(x,k,\beta \right)\\ \mp \frac{\Phi_{F}}{{\left(2\pi \right)}^{d}}\int_{R^{2}}w_{eq}\left(x,k^{\prime },\beta \right)w_{eq}\left(x,k^{\prime }-k,\beta \right)dk^{\prime }+o\left(\hbar^{2}\right). \end{array}$$

Of course the general solution is still difficult to get analytically. However, in order to compare the results with those already known in the literature, in the next section particular cases will be considered where explicit analytical solutions are found.

## 5. Particular Cases

Let us consider the homogeneous case. We have Φ(x)=Φ=constant and the Wigner function does not depend on x, that is w=w(k,β). Under these assumption the Equation (10) reads

(13) $$\begin{array}{ccc} \frac{\partial w_{eq}\left(k,\beta \right)}{\partial \beta }= & - & \varepsilon \left(k\right)w_{eq}\left(k,\beta \right)-\Phi w_{eq}\left(k,\beta \right)\pm \frac{1}{{\left(2\pi \right)}^{d}}\;\varepsilon \left(k\right)\int_{R^{2}}w_{eq}\left(k^{\prime },\beta \right)w_{eq}\left(k^{\prime }-k,\beta \right)dk^{\prime }\\ & \pm & \frac{q\Phi }{{\left(2\pi \right)}^{d}}\int_{R^{2}}w_{eq}\left(k^{\prime },\beta \right)w_{eq}\left(k^{\prime }-k,\beta \right)dk^{\prime }+\Phi_{F}\;w_{eq}\left(k,\beta \right)\\ & \mp & \frac{\Phi_{F}}{{\left(2\pi \right)}^{d}}\int_{R^{2}}w_{eq}\left(k^{\prime },\beta \right)w_{eq}\left(k^{\prime }-k,\beta \right)dk^{\prime }.\; \end{array}$$

To solve this equation let us consider the Laplace transform of the equilibrium Wigner function

$$\hat{w}\left(k,s\right)=\int_{0}^{+\infty }e^{-s\beta }w_{eq}\left(k,\beta \right)d\beta .$$

By taking the Laplace transform of the Equation (13), one obtains

(14) $$s\;\hat{w}\left(k,s\right)-w_{eq}\left(k,0^{+}\right)=-\left(\varepsilon +\Phi -\Phi_{F}\right)\hat{w}\left(k,s\right)\pm \frac{1}{{\left(2\pi \right)}^{d}}\left(\varepsilon +\Phi -\Phi_{F}\right){\hat{w}}^{2}\left(k,s\right)$$

augmented with the condition $w_{eq}\left(k,0^{+}\right)=1$.

Now we treat separately the case of fermions and bosons.

### 5.1. Fermions

Equation (14) gives

$$\frac{1}{{\left(2\pi \right)}^{d}}\left(\varepsilon +\Phi -\Phi_{F}\right){\hat{w}}^{2}\left(k,s\right)-\left(s+\varepsilon +\Phi -\Phi_{F}\right)\hat{w}\left(k,s\right)+1=0$$

whose solutions are

$${\hat{w}}_{\pm }\left(k,s\right)=\frac{{\left(2\pi \right)}^{d}}{2}\frac{\left(s+\varepsilon +\Phi -\Phi_{F}\right)\pm \sqrt{{\left(s+\varepsilon +\Phi -\Phi_{F}\right)}^{2}-\frac{4}{{\left(2\pi \right)}^{d}}\left(\varepsilon +\Phi -\Phi_{F}\right)}}{\varepsilon +\Phi -\Phi_{F}}$$

By taking the inverse Laplace transform, we get

(15) $$\begin{array}{c} w_{+}\left(k,\beta \right)=-\frac{e^{-\beta (\varepsilon +\Phi -\Phi_{F})}}{\beta \sqrt{\frac{\varepsilon +\Phi -\Phi_{F}}{{\left(2\pi \right)}^{d}}}}I_{1}\left(2\beta \sqrt{\frac{\varepsilon +\Phi -\Phi_{F}}{{\left(2\pi \right)}^{d}}}\right)+\delta \left(\beta \right)+\frac{1}{\varepsilon +\Phi -\Phi_{F}}\delta^{\prime }\left(\beta \right),\\ w_{-}\left(k,\beta \right)=\frac{e^{-\beta (\varepsilon +\Phi -\Phi_{F})}}{\beta \sqrt{\frac{\varepsilon +\Phi -\Phi_{F}}{{\left(2\pi \right)}^{d}}}}I_{1}\left(2\beta \sqrt{\frac{\varepsilon +\Phi -\Phi_{F}}{{\left(2\pi \right)}^{d}}}\right) \end{array}$$

where $I_{n}\left(x\right)$ is the modified Bessel function of first kind of order *n*.

Of course, the first solution is not classical but valid in the distributional sense. We disregard it looking as unphysical. The second solution is a kind of modulation of the Wigner function in the case of the von Neumann quantum entropy, already found in [24],

$$w^{*}=e^{-\beta (\varepsilon +\Phi -\Phi_{F})}.$$

which, of course, is a classical distribution if $\varepsilon +\Phi \geq \Phi_{F}$.

We recall the serie expansion of $I_{1}\left(z\right)$, valid for every *z* in the complex plane [25],

(16) $$I_{1}\left(z\right)=\frac{1}{2}z\sum_{k=0}^{+\infty }\frac{{\left(\frac{1}{4}z^{2}\right)}^{k}}{k!\Gamma (k+2)}$$

with Γ(z) Euler’ s gamma function.

From (16) it is easy verify that (15) tends to 1 as $\beta \mapsto 0^{+}$. Moreover, if *z* is a pure imaginary complex number then $I_{1}\left(z\right)$ is also a pure imaginary complex number. Therefore, by choosing for the square root the branch $\sqrt{ix}=i\sqrt{\left|x\right|}$ for any x∈R, the Wigner function (15) is real.

### 5.2. Bosons

In this case Equation (14) gives

$$\frac{1}{{\left(2\pi \right)}^{d}}\left(\varepsilon +\Phi -\Phi_{F}\right){\hat{w}}^{2}\left(k,s\right)+\left(s+\varepsilon +\Phi -\Phi_{F}\right)\hat{w}\left(k,s\right)-1=0$$

whose solutions are

$${\hat{w}}_{\pm }\left(k,s\right)=\frac{{\left(2\pi \right)}^{d}}{2}\frac{-\left(s+\varepsilon +\Phi -\Phi_{F}\right)\pm \sqrt{{\left(s+\varepsilon +\Phi -\Phi_{F}\right)}^{2}+\frac{4}{{\left(2\pi \right)}^{2}}\left(\varepsilon +\Phi -\Phi_{F}\right)}}{\varepsilon +\Phi -\Phi_{F}}$$

By taking the inverse Laplace transform, we get

(17) $$\begin{array}{c} w_{+}\left(k,\beta \right)=\frac{e^{-\beta (\varepsilon +\Phi -\Phi_{F})}}{\beta \sqrt{-\frac{\varepsilon +\Phi -\Phi_{F}}{{\left(2\pi \right)}^{d}}}}I_{1}\left(2\beta \sqrt{-\frac{\varepsilon +\Phi -\Phi_{F}}{{\left(2\pi \right)}^{d}}}\right),\\ w_{-}\left(k,\beta \right)=-\frac{e^{-\beta (\varepsilon +\Phi -\Phi_{F})}}{\beta \sqrt{-\frac{\varepsilon +\Phi -\Phi_{F}}{{\left(2\pi \right)}^{d}}}}I_{1}\left(2\beta \sqrt{-\frac{\varepsilon +\Phi -\Phi_{F}}{{\left(2\pi \right)}^{d}}}\right)-\delta \left(\beta \right)-\frac{1}{\varepsilon +\Phi -\Phi_{F}}\delta^{\prime }\left(\beta \right) \end{array}$$

As in the previous case the second solution is not classical but valid in the distributional sense and we disregard it looking as unphysical. The first solution is again a kind of modulation of the Wigner function in the case of the von Neumann quantum entropy. Note that when the argument of

$$\frac{1}{\beta \sqrt{-\frac{\varepsilon +\Phi -\Phi_{F}}{{\left(2\pi \right)}^{d}}}}I_{1}\left(2\beta \sqrt{-\frac{\varepsilon +\Phi -\Phi_{F}}{{\left(2\pi \right)}^{d}}}\right)$$

is imaginary, the analogous for the (15) is real and vice versa.

We stress that both (15) and (17) are valid for any dispersion relation.

**Figure 1:**
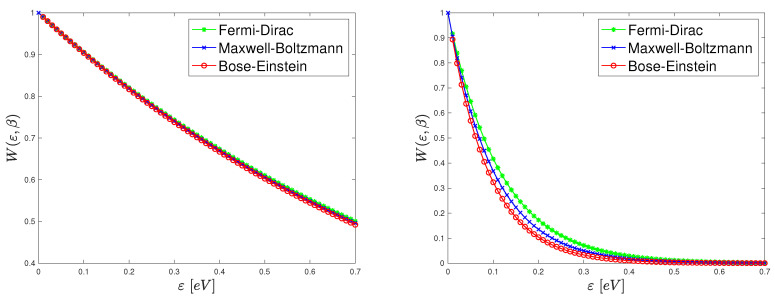

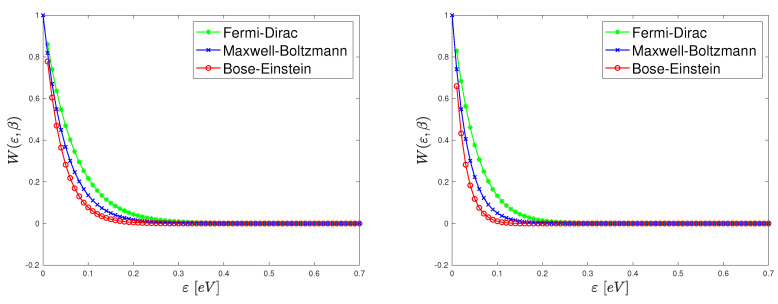
Plots of the equilibrium Wigner function versus energy (in eV) for several values of the parameter β: 1 (**left top**), 10 (**right top**), 20 (**left bottom**), 30 (**right bottom**) 1/eV.

In Figure 1 the Wigner functions (15) and (17) are plotted versus energy for several values of the parameter β: 1, 10, 20, 30 eV${}^{-1}$. Note that at room temperature (300 K) one has β≈38.61 eV${}^{-1}$. For comparison, $w^{*}$ is also shown. It is evident that by increasing the temperature, that is by decreasing β, the degeneracy effect becomes negligible and $w^{*}$ and the equilibrium Wigner functions (17) and (15) tend to coincide. This is in according with the general results in statistical mechanics but here it has been deduced, to the best of our knowledge for the first time, from an analysis of the equilibrium Wigner function including Boson and Fermion statistics. At low temperature relevant differences are obtained between the degenerate and non degenerate cases. This indicates that the use of the standard von Neumann entropy leads to equilibrium Wigner functions which are not suited for a correct description of physical problems involving the transport of fermions and bosons. Observe that the Wigner function is not always positive definite because of the Heisenberg uncertainty principle. However, in the homogeneous case here considered the position is completely uncertain so the momentum is certain and the Wigner function is positive. Therefore, after a suitable normalization, it can be regarded as a classical distribution. Although apparently simple, the case of constant potential has physically relevant applications. If one considers the transport of phonons in a crystal lattice without any mechanical deformation, they do not undergo any external field but have a dispersion relation which is not usually quadratic. For example acoustic phonons have a linear dispersion relation near the center of the first Brillouin zone (the Debye approximation), that is

$$\varepsilon \left(p\right)=c_{s}\hbar \left|p\right|$$

where $c_{s}$ is the sound speed.

## 6. Conclusions

By considering an expression of the quantum entropy which takes also into account the Fermi and the Bose statistics, a general equation for the equilibrium Wigner function, valid for any energy dispersion relation, has been deduced. Particular solutions have been obtained in the spacial homogeneous case, generalizing what already known in the literature by assuming as quantum entropy that proposed by von Neumann.

To find explicit analytical solutions of Equation (10) in the general case is a daunting task. As open problem it should be interesting to develop appropriate numerical schemes to solve the Equation (10) or its version up to first order in $\hbar^{2}$, Equation (12). A viable way could be to modify the approach in [26]. The equilibrium Wigner function can be used into the relaxation time approximation of the collision term in the Wigner–Boltzmann equation or to introduce quantum corrections in the description of quantum fluids, e.g., to existing drift-diffusion, energy-transport and hydrodynamical models for charge transport. A possible application could be a generalization of the model for electron transport in graphene presented in [27]. We mention also that in view of the enhancement of the importance of the thermal effects in nanoscale devices, the developed formalism can be also adopted for a quantum description of phonon transport based on the Wigner equation.

## Appendix A

Here the derivation of the Wigner Transport equation is recalled for a general energy dispersion relation. We have to specify the meaning of the operator $E(-i\hbar \nabla_{r})$. The operator $E\left(-i\hbar \nabla_{r}\right)\rho \left(r,s,t\right)$ is defined as a multiplication operator in the Fourier transform space and then mapped back in the **r**-space

(A1) $$E\left(-i\hbar \nabla_{r}\right)\rho \left(r,s,t\right)=\frac{1}{{\left(2\pi \hbar \right)}^{d}}\int_{R_{\eta }^{d}\times R_{r^{\prime }}^{d}}E\left(\eta \right)\rho \left(r^{\prime },s,t\right)e^{-i\frac{\eta }{\hbar }\cdot \left(r^{\prime }-r\right)}d\eta dr^{\prime }.$$

where η is the momentum conjugate to $r^{\prime }$.

Now we are denoting with p the momentum conjugate with y. Using the change of coordinates

$$r=x+\frac{y}{2},\;s=x-\frac{y}{2},$$

and after observing that

$$x=\frac{r+s}{2},\;y=r-s,$$

the expressions of $\nabla_{r}$ and $\nabla_{s}$ are

$$\begin{array}{c} \nabla_{r}=\frac{1}{2}\nabla_{x}+\nabla_{y},\;\nabla_{s}=\frac{1}{2}\nabla_{x}-\nabla_{y}, \end{array}$$

and the symbols associated to $E(-i\hbar \nabla_{r})$ and $E(-i\hbar \nabla_{s})$ become

$$\begin{array}{c} E\left(p+\frac{1}{2}\eta \right),\;E\left(p-\frac{1}{2}\eta \right), \end{array}$$

respectively, where the fact that E is an even function has been used.

If we set $u\left(x,y,t\right):=\rho \left(x+\frac{y}{2},x-\frac{y}{2},t\right)$ then w=F[u], where the Fourier transform is respect to the variable y, and of course

(A2) $$u\left(x,y,t\right)=F^{-1}\left[w\right]=\frac{1}{{\left(2\pi \hbar \right)}^{d}}\int_{R_{p}^{d}}w\left(x,p,t\right)e^{ip\cdot y/\hbar }dp.$$

Fourier transforming Equation (1) gives

$$i\hbar \frac{\partial }{\partial t}F\left[u\right]\left(x,p,t\right)=F\left[\left(E\left(p+\frac{1}{2}\eta \right)-E\left(p-\frac{1}{2}\eta \right)\right)u\left(x,y,t\right)+\left[(\Phi (r,t)-\Phi (s,t))\right]u\left(x,y,t\right)\right]\left(x,p,t\right).$$

From (A1) and (4), one has

(A3) $$\begin{array}{c} F\left[\left(E\left(p+\frac{1}{2}\eta \right)-E\left(p-\frac{1}{2}\eta \right)\right)u\left(x,y,t\right)\right]\left(x,p,t\right)=\\ =\frac{1}{{\left(2\pi \hbar \right)}^{d}}F\left[\int_{R_{x^{\prime }}^{d}\times R_{\eta }^{d}}\left(E\left(p+\frac{1}{2}\eta \right)-E\left(p-\frac{1}{2}\eta \right)\right)u\left(x^{\prime },y,t\right)e^{i\eta \cdot \frac{\left(x-x^{\prime }\right)}{\hbar }}d\eta dx^{\prime }\right]=\\ =\frac{1}{{\left(2\pi \hbar \right)}^{d}}\int_{R_{x^{\prime }}^{d}\times R_{\eta }^{d}\times R_{y}^{d}}\left(E\left(p+\frac{1}{2}\eta \right)-E\left(p-\frac{1}{2}\eta \right)\right)u\left(x^{\prime },y,t\right)e^{i\eta \cdot \frac{\left(x-x^{\prime }\right)}{\hbar }}\;e^{-ip\cdot y/\hbar }\;d\eta \;dx^{\prime }\;dy=\\ =\frac{1}{{\left(2\pi \hbar \right)}^{d}}\int_{R_{x^{\prime }}^{d}\times R_{\eta }^{d}}\left(E\left(p+\frac{1}{2}\eta \right)-E\left(p-\frac{1}{2}\eta \right)\right)w\left(x^{\prime },p,t\right)e^{i\eta \cdot \frac{\left(x-x^{\prime }\right)}{\hbar }}\;\;d\eta \;dx^{\prime }, \end{array}$$

and

(A4) $$\begin{array}{c} F\left[\left(\Phi \left(x+\frac{y}{2}\right)-\Phi \left(x-\frac{y}{2}\right)\right)u\left(x,y,t\right)\right]\left(x,p,t\right)=\\ =\frac{1}{{\left(2\pi \hbar \right)}^{d}}F\left[\int_{R_{p^{\prime }}^{d}}\left(\Phi \left(x+\frac{y}{2}\right)-\Phi \left(x-\frac{y}{2}\right)\right)w\left(x,p^{\prime },t\right)e^{ip^{\prime }\cdot y/\hbar }dp^{\prime }\right]\;=\\ =\frac{1}{{\left(2\pi \hbar \right)}^{d}}\int_{R_{y}^{d}\times R_{p^{\prime }}^{d}}\left(\Phi \left(x+\frac{y}{2}\right)-\Phi \left(x-\frac{y}{2}\right)\right)w\left(x,p^{\prime },t\right)e^{i(p^{\prime }-p)\cdot y/\hbar }\;dp^{\prime }\;dy. \end{array}$$

Altogether, the Wigner function satisfies the equation

$$\frac{\partial w(x,p,t)}{\partial t}+S\left[E\right]w\left(x,p,t\right)+\theta \left[E\right]w\left(x,p,t\right)=0.$$

with S[E] and θ[E] definited in (3)–(4).

A further generalization includes also the presence of a sort of collision term into the Wigner function obtaining (2).

Approximating with the Taylor expansion centered at ℏ=0 we get

(A5) $$E\left(p+\frac{\hbar }{2}\nu ,t\right)-E\left(p-\frac{\hbar }{2}\nu ,t\right)=\nabla_{p}E\left(p\right)\cdot \hbar \nu +\frac{1}{24}\frac{\partial^{3}E\left(p\right)}{\partial p_{i}p_{j}p_{k}}\hbar^{3}\nu_{i}\nu_{j}\nu_{k}+O\left(\hbar^{5}\right),$$

(A6) $$\Phi \left(x+\frac{\hbar }{2}\eta ,t\right)-\Phi \left(x-\frac{\hbar }{2}\eta ,t\right)=\nabla_{x}\Phi \left(x\right)\cdot \hbar \eta +\frac{1}{24}\frac{\partial^{3}\Phi \left(x\right)}{\partial x_{i}\partial x_{j}\partial x_{k}}\hbar^{3}\eta_{i}\eta_{j}\eta_{k}+O\left(\hbar^{5}\right).$$

Substituting the previous relations into (3) and (4) we obtain

(A7) $$\begin{array}{cc} S[E]w(x,p,t)\approx & \frac{i}{\hbar {\left(2\pi \right)}^{d}}\int_{R_{x^{\prime }}^{d}\times R_{\nu }^{d}}\left[\nabla_{p}E\left(p\right)\cdot \hbar \nu +\frac{1}{24}\frac{\partial^{3}E\left(p\right)}{\partial p_{i}\partial p_{j}\partial p_{k}}\hbar^{3}\nu_{i}\nu_{j}\nu_{k}\right]w\left(x^{\prime },p,t\right)e^{-i(x^{\prime }-x)\cdot \nu }dx^{\prime }d\nu \\ & =\frac{i}{{\left(2\pi \right)}^{d}}\nabla_{p}E\left(p\right)\cdot \int_{R_{x^{\prime }}^{d}\times R_{\nu }^{d}}\nu w\left(x^{\prime },p,t\right)e^{-i(x^{\prime }-x)\cdot \nu }dx^{\prime }d\nu \\ & +\frac{i}{{\left(2\pi \right)}^{d}}\frac{1}{24}\frac{\partial^{3}E\left(p\right)}{\partial p_{i}\partial p_{j}\partial p_{k}}\hbar^{2}\int_{R_{x^{\prime }}^{d}\times R_{\nu }^{d}}\nu_{i}\nu_{j}\nu_{k}w\left(x^{\prime },p,t\right)e^{-i(x^{\prime }-x)\cdot \nu }dx^{\prime }d\nu \\ & =\nabla_{p}E\left(p\right)\cdot \nabla_{x}w\left(x,p,t\right)-\frac{\hbar^{2}}{24}\frac{\partial^{3}E\left(p\right)}{\partial p_{i}\partial p_{j}\partial p_{k}}\frac{\partial^{3}w\left(x,p,t\right)}{\partial x_{i}\partial x_{j}\partial x_{k}} \end{array}$$

and

(A8) $$\begin{array}{cc} \theta [E]w(x,p,t)\approx & \frac{i}{\hbar {\left(2\pi \right)}^{d}}\int_{R_{p^{\prime }}^{d}\times R_{\;\eta }^{d}}\left[\nabla_{x}\Phi \left(x\right)\cdot \hbar \eta +\frac{1}{24}\frac{\partial^{3}\Phi \left(x\right)}{\partial x_{i}\partial x_{j}\partial x_{k}}\hbar^{3}\eta_{i}\eta_{j}\eta_{k}\right]w\left(x,p^{\prime },t\right)e^{i(p^{\prime }-p)\cdot \eta }dp^{\prime }d\eta \\ & =-\nabla_{x}\Phi \left(x\right)\cdot \nabla_{p}w\left(x,p,t\right)+\frac{\hbar^{2}}{24}\frac{\partial^{3}\Phi \left(x\right)}{\partial x_{i}\partial x_{j}\partial x_{k}}\frac{\partial^{3}w\left(x,p,t\right)}{\partial p_{i}\partial p_{j}\partial p_{k}}. \end{array}$$
