# Correction: Camiola, V.D., et al. Equilibrium Wigner Function for Fermions and Bosons in the Case of a General Energy Dispersion Relation. *Entropy* 2020, *22*, 1023

**DOI:** 10.3390/e23040417

**Published:** 2021-03-31

**Authors:** Vito Dario Camiola, Liliana Luca, Vittorio Romano

**Affiliations:** Department of Mathematics and Computer Science, University of Catania, 95125 Catania, Italy; lilianaluca88@gmail.com (L.L.); romano@dmi.unict.it (V.R.)

In Section 5 of *Equilibrium Wigner Function for Fermions and Bosons in the Case of a General Energy Dispersion Relation* (*Entropy* **2020**, *22*, 1023 [1]), the Laplace transform was used in the wrong way to find the solution of Equation (13); therefore, the derived Equation (14) is not correct. Here, we rewrite Section 5, the determination of examples of the solution by adopting suitable asymptotic expansions. This correction does not modify the main conclusion of the article, except the fact that we find additional solutions that do not represent only a modulation of the solutions in the non-degenerate case.

The authors apologize for the oversight and hope that it has not had affected the work of other colleagues.

The new section is as follows.

## 5. Particular Cases

Let us consider the homogeneous case. We have Φ(x)=Φ=constant, and the Wigner function does not depend on x, that is w=w(k,β). Moreover, we assume that the first Brillouin zone is finite, that is, $w_{eq}\left(k,\beta \right)$ is zero outside a compact set B, which is symmetric with respect to the origin. Under these assumption, Equation (10) reads

(13) $$\begin{array}{ccc} \frac{\partial w_{eq}\left(k,\beta \right)}{\partial \beta }= & - & \left(\varepsilon \left(k\right)+\Phi -\Phi_{F}\right)w_{eq}\left(k,\beta \right)+\\ & \pm & \frac{\varepsilon \left(k\right)+\Phi -\Phi_{F}}{{\left(2\pi \right)}^{d}}\int_{B}w_{eq}\left(k^{\prime },\beta \right)w_{eq}\left(k^{\prime }-k,\beta \right)dk^{\prime }. \end{array}$$

Solving this equation is a very difficult task, and here, we propose two possible ways to find an approximate solution that satisfies the condition $\lim_{\beta \to 0^{+}}w_{eq}\left(k,\beta \right)=1$ (see [24]). We assume the ansatz

$$w_{eq}\left(k,\beta \right)=\left(1+\sum_{m=1}^{n}\lambda_{m}\left(k\right)\beta^{m}\right)e^{-\beta \varepsilon (k)}$$

with n∈N and λ:B→R.

Substituting in (13) and considering the lower order terms, one obtains

$$\lambda_{1}\left(k\right)=\phi_{F}-\phi \pm \frac{\varepsilon \left(k\right)+\phi -\phi_{F}}{{\left(2\pi \right)}^{d}}\mu \left(B\right)$$

where μ(B) is the measure of the first Brillouin zone. The equilibrium Wigner function approximated at the first order reads

$$w_{eq}\left(\beta ,k\right)=\left[1+\left(\phi_{F}-\phi \pm \frac{\varepsilon \left(k\right)+\phi -\phi_{F}}{{\left(2\pi \right)}^{d}}\mu \left(B\right)\right)\beta \right]e^{-\beta \varepsilon (k)}+o\left(\beta \right).$$

The other terms $\lambda_{m}\left(k\right)$ with m≥2 can be found with an iterative procedure.

Another analytical result can be found around the origin of the first Brillouin zone, that is, for |k|≪1. About the dispersion relation, the natural assumption $\varepsilon \left(k\right)=ak^{2}+{o(|k|}^{2})$, with $a\in R^{+}$, is made for |k|≪1.

We look for solutions according to the ansatz $w_{eq}\left(k,\beta \right)=\gamma \left(k,\beta \right)e^{-\beta \varepsilon (k)}$ with γ(k,β), a slowly varying function with respect to k: $\gamma \left(k,\beta \right)\approx \gamma_{0}\left(\beta \right)+\gamma_{1}\left(\beta \right)o\left(|k|\right)$.

By substituting in (13), one obtains

(14) $$\begin{array}{c} \frac{d\gamma_{0}\left(\beta \right)}{d\beta }+\frac{d\gamma_{1}\left(\beta \right)}{d\beta }o\left(|k|\right)=\left(\phi_{F}-\phi \right)\left(\gamma_{0}\left(\beta \right)+\gamma_{1}\left(\beta \right)o\left(|k|\right)\right)\\ \pm \frac{e^{\beta \varepsilon (k)}}{{\left(2\pi \right)}^{2}}\left(\varepsilon \left(k\right)+\phi -\phi_{F}\right)\int_{B}{\left[\gamma_{0}\left(\beta \right)+\gamma_{1}\left(\beta \right)o\left(|k|\right)\right]}^{2}e^{-\beta \left(\varepsilon \left(k^{\prime }\right)+\varepsilon \left(k^{\prime }-k\right)\right)}dk^{\prime }. \end{array}$$

At the lowest order, we have

(15) $$\begin{array}{c} \frac{d\gamma_{0}\left(\beta \right)}{d\beta }=\left(\phi_{F}-\phi \right)\gamma_{0}\left(\beta \right)\pm \frac{1}{{\left(2\pi \right)}^{2}}\left(\phi -\phi_{F}\right){\left[\gamma_{0}\left(\beta \right)\right]}^{2}\int_{B}e^{-2a\beta k^{\prime }{|}^{2}}\left(1-2a\beta k^{\prime }\cdot k)\right)\;dk^{\prime }. \end{array}$$

Due to the symmetry of B

$$\int_{B}e^{-2a\beta k^{\prime }{|}^{2}}k^{\prime }\cdot k\;dk^{\prime }=0$$

and, therefore, $\gamma_{0}\left(\beta \right)$ satisfies the equation

(16) $$\begin{array}{c} \frac{d\gamma_{0}\left(\beta \right)}{d\beta }=\left(\phi_{F}-\phi \right)\gamma_{0}\left(\beta \right)\pm \frac{1}{{\left(2\pi \right)}^{2}}\left(\phi -\phi_{F}\right){\left[\gamma_{0}\left(\beta \right)\right]}^{2}\int_{B}e^{-2a\beta k^{\prime }{|}^{2}}\;dk^{\prime } \end{array}$$

for which the solution is

$$\gamma \left(\beta \right)=\frac{e^{\beta (\phi_{F}-\phi )}}{1\mp \int_{0}^{\beta }\alpha \left(\beta^{\prime }\right)e^{\beta^{\prime }\left(\phi_{F}-\phi \right)}d\beta^{\prime }}$$

with

$$\alpha \left(\beta \right)=\frac{\left(\phi -\phi_{F}\right)}{{\left(2\pi \right)}^{d}}\int_{B}e^{-2a\beta k^{\prime }{|}^{2}}\;dk^{\prime }.$$

Therefore, the equilibrium Wigner function around the center of the first Brillouin zone is

(17) $$w_{eq}\left(k,\beta \right)=\frac{e^{-\beta \left(\varepsilon \left(k\right)+\phi -\phi_{F}\right)}}{1\mp \int_{0}^{\beta }\alpha \left(\beta^{\prime }\right)e^{\beta^{\prime }\left(\phi_{F}-\phi \right)}d\beta^{\prime }}.$$

In order to present some numerical results, let us consider a tridimensional gas of non-interacting particles with a quadratic dispersion relation $\varepsilon \left(k\right)=ak^{2}$, $a\in R^{+}$. To analytically evaluate the term α(β), B is extended to $R^{3}$, obtaining

$$\alpha \left(\beta \right)=\sqrt{\frac{\pi }{2a\beta }}\frac{\left(\phi -\phi_{F}\right)}{{\left(2\pi \right)}^{2}},$$

which, inserted into (17), gives

(18) $$w_{eq}\left(\varepsilon ,\beta \right)=\frac{e^{-\beta (\varepsilon +\phi -\phi_{F})}}{1\mp \sqrt{\frac{\pi }{2a}}\frac{\phi -\phi_{F}}{4\pi^{2}}\int_{0}^{\beta }\frac{1}{\sqrt{\beta^{\prime }}}e^{-\beta^{\prime }\left(\phi -\phi_{F}\right)}d\beta^{\prime }}.$$

In Figure 1, the Wigner function (18) is plotted versus energy for several values of the parameters β and $\phi -\phi_{F}$. For comparison, the Maxwell–Boltzmann distribution $w^{*}=e^{-\beta (\varepsilon +\phi -\phi_{F})}$ is also shown. It is important to observe that, at high temperatures, that is, low β’s, the equilibrium Wigner functions for Bosons and Fermions are both close to the Maxwell–Boltzmann distribution; therefore, Bosons and Fermions tend to have the same behaviour for $\beta \to 0^{+}$. Moreover, in the plotted cases, the equilibrium Wigner function is positive. At low temperatures (high values of β), the behaviour strongly depends on $\phi -\phi_{F}$. When $\phi -\phi_{F}=0.5$, the equilibrium Wigner functions for Bosons and Fermions are still very close and positive. If $\phi -\phi_{F}=-0.5$, the equilibrium Wigner functions of Bosons and Fermions and the Maxwell–Boltzmann distribution have relevant differences: the most remarkable one is that the Fermions Wigner function is negative while the others are positive.

As a last remark, although apparently simple, the case of constant potential has physically relevant applications. If one considers the transport of phonons in a crystal lattice without any mechanical deformation, they do not undergo any external field but have a dispersion relation that is not usually quadratic. For example, acoustic phonons have a linear dispersion relation near the center of the first Brillouin zone (the Debye approximation), that is

$$\varepsilon \left(p\right)=c_{s}\hbar \left|p\right|$$

where $c_{s}$ is the sound speed.

## Figures and Tables

**Figure 1:**
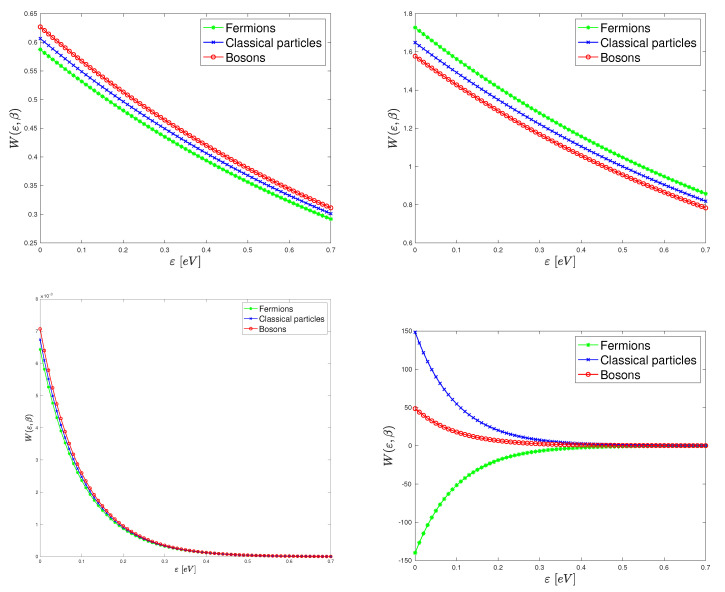
Plots of the equilibrium Wigner function versus energy (in eV) for several values of the parameters β and $\phi -\phi_{F}$. **Left top**: β=1, $\phi -\phi_{F}=0.5$. **Right top**: β=1, $\phi -\phi_{F}=-0.5$. **Left bottom**: β=10, $\phi -\phi_{F}=0.5$. **Right bottom**: β=10, $\phi -\phi_{F}=-0.5$. We set a=0.7. Arbitrary units are used.
